# A Hybrid Antenna Array Design for 3-D Direction of Arrival Estimation

**DOI:** 10.1371/journal.pone.0118914

**Published:** 2015-03-19

**Authors:** Najam-Us Saqib, Imdad Khan

**Affiliations:** COMSATS Institute of Information Technology, Abbottabad, Pakistan; University of Chinese Academy of Sciences, CHINA

## Abstract

A 3-D beam scanning antenna array design is proposed that gives a whole 3-D spherical coverage and also suitable for various radar and body-worn devices in the Body Area Networks applications. The Array Factor (AF) of the proposed antenna is derived and its various parameters like directivity, Half Power Beam Width (HPBW) and Side Lobe Level (SLL) are calculated by varying the size of the proposed antenna array. Simulations were carried out in MATLAB 2012b. The radiators are considered isotropic and hence mutual coupling effects are ignored. The proposed array shows a considerable improvement against the existing cylindrical and coaxial cylindrical arrays in terms of 3-D scanning, size, directivity, HPBW and SLL.

## Introduction

Antenna arrays play a vital role in modern wireless communication systems. Normally, single antenna element provides a relatively wide radiation pattern, having low values of directivity/gain [1]. In numerous applications, it is desirable to design the antennas with highly directive characteristics in order to meet the hassles of long distance and interference free communication. In order to achieve the required performance, antenna array is used. Due to the increase in demand for capacity in wireless communication, the innovations in deploying smart antenna systems are being made now-a-days [2]. By getting the narrower main radiation beam and nulls in the undesired directions, the issue of getting more Signal of Interest (SoI) and simultaneously rejecting the Signals Not of Interest (SNoI) can be resolved.

Antenna array geometry considerably affects the main lobe characteristics and the beam scanning capabilities of the array. The geometry may be 1-D, 2-D or 3-D depending upon our requirements. The increase in dimension improves the capability of scanning the main radiation beam. Among the 2-D geometries, circular array is of greater importance as the element arrangements are in continuous pattern and hence, we do not have any edges or discontinuities among the radiators. This provides an advantage of electronic rotation of the plane of the circular array without altering the shape of main beam [3]. The tradeoff in the circular array is that it has high side lobe level and it does not have any nulls in the azimuth plane, which is a necessary feature in the smart antenna systems to reject SNoI [1]. The SLL can be reduced by using concentric circular array geometry [4]. Concentric circular array has several advantages over that of circular array which includes their efficient use in both broadband and narrowband beamforming [5]. The effects of various antenna array geometries like uniform linear, circular, rectangular and cubic on multiple-input multiple-output channels are investigated in [6] which shows that in various propagation scenarios, uniform linear array show superiority in terms of ergodic channel capacity in azimuth orientation. A beam scanning method using mechanical motion of elements of the circular array is presented in [7]. The drawback in the design is that it was using the physical movements of the elements to acquire the required scanning. In [8], radiation properties of a circular array of centrally fed dipole radiators are analyzed that shows its better performance in terms of directivity, gain and also full 360^0^ azimuth scanning. An elliptical array of end fed elements with a main beam towards normal direction of the array is discussed in [9]. Another method of reducing the SLL by using concentric circular array is presented in [10]. Here, concentric circular array was proved to be the best candidate for sidelobe level reduction.

A hybrid combination of linear and circular arrays is discussed in [11]. Moreover, the combined properties of uniform linear and uniform circular antenna arrays are discussed. Directivity, half power beamwidth and sidelobe level of the proposed design are calculated and compared. Different types of hybrid elliptical arrays are presented in [12] and various 3-D geometries are discussed and compared. The advantages of 3-D geometries are also presented. In [13], a method of designing concentric circular and concentric hexagonal array design is presented. Different aspects of concentric circular and hexagonal array have been discussed. Moreover, their advantages over other planar geometries are also presented. The characteristics and comparisons between spherical and cylindrical arrays are discussed in [14]. Spherical arrays have complex geometry but have super directive properties, whereas, the cylindrical arrays offer simpler geometry that can easily be implemented.

This research work is focused on the general shape (geometry) of the antenna array, as its geometry has a significant effect on the radiation pattern characteristics. Moreover, different parameters of the antenna arrays that are necessary for Direction of Arrival (DOA) and beamforming applications are also considered in this paper. Various properties of linear, circular, concentric circular and cylindrical arrays, as discussed above, are combined and a new hybrid 3-D antenna array geometry is presented that provides a uniform scanning in 3-D sphere. The antenna elements used are isotropic and hence no mutual coupling is considered. Amplitude excitations are considered uniform in order to reduce overhead. Various characteristics of the proposed array are presented by varying different design parameters. The deficiencies provided by the previous antenna array geometries in terms of uniform directivity in 3D space for circular, cylindrical and concentric cylindrical arrays, HPBW and high SLL or high grating lobe can be reduced by using the proposed model.

The rest of the paper is organized into four sections as follows: In Section II, the geometry of the proposed antenna array is presented. In Section III, different simulation results and comparisons are reported. Finally, conclusions are given in Section IV.

### Proposed Array

#### Array Geometry

**Fig 1:**
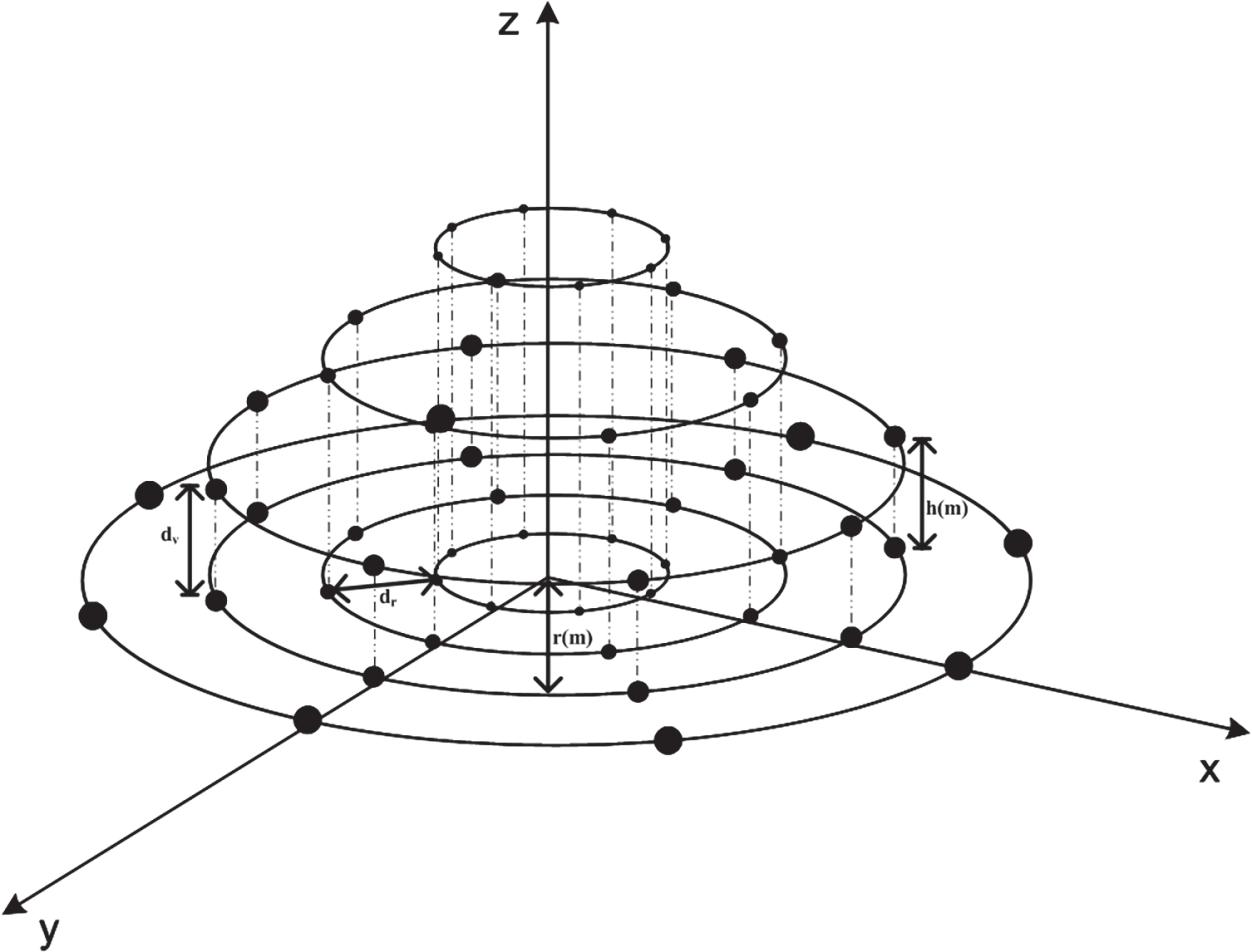
Proposed Array Geometry.

The proposed antenna structure is a combination of different circular, linear and cylindrical arrays. The design consists of 2M + 1 circular rings of elements. Out of which, M+1 circular rings lie on the x-y plane and other M rings are stacked above the x-y plane. A 7-ring array design is shown in Fig. 1. In the proposed structure, we have a total of M cylindrical arrays along with a circular ring lying on the x-y plane, where each cylindrical array comprises of exactly two circular arrays stacked upon each other, as shown in Fig. 1. The inter-ring spacings, *d*_*v*_ and *d*_*r*_, on the vertical and traversal plane, respectively, are taken as half wavelength. In Fig. 1, h_m_ is the vertical height between two rings of the m^th^ cylindrical array, r_m_ is radius of the rings of m^th^ cylindrical array, d_v_ is vertical spacing between two consecutive rings and d_r_ is horizontal spacing between two consecutive rings.

#### Array Factor of the Proposed Geometry

By observing the design closely, we see that a proper symmetry lies in it. Every circular ring at the x-y plane (*z* = 0) has its duplicate, which exists (separated by some height *h*_*m*_) above that plane; except the outer most circular ring. The circular ring pairs form different 2-ring cylindrical arrays. Hence, we have exactly *M* cylindrical arrays and one outer most circular ring that lies in the x-y plane.

The array factor of the cylindrical array is given by [11]:

$${AF}_{cyl}=\sum_{p=1}^{p}\sum_{n=1}^{N}C_{\mathrm{np}}e^{jkr\sin\theta \cos(\emptyset -\emptyset_{n})}$$ (1)

where

$$C_{np}=I_{n}e^{\alpha_{n}}b_{p}e^{j\left(p-1\right)(kdcos\theta +\beta )}$$

where *P* is the number of circular rings in the cylinder and *N* is the total number of elements in a circular ring. As every cylindrical array in this case consists of two rings, therefore, the array factor will be simplified to *P* = 2:

$$AF_{cyl}=\sum_{n=1}^{N}\left[C_{n1}e^{jkrsin\theta \cos\left(\emptyset -\emptyset_{n}\right)}+C_{n2}e^{jkrsin\theta \cos\left(\emptyset -\emptyset_{n}\right)}\right]$$ (2)

Putting the values of *C*_*n*1_ and *C*_*n*2_ in the above expression we get:

$$AF_{cyl}=\sum_{n=1}^{N}\left[I_{n}e^{\alpha_{n}}b_{1}e^{jkrsin\theta cos\left(\emptyset -\emptyset_{n}\right)}+I_{n}e^{\alpha_{n}}b_{2}e^{j\left(kdcos\theta +\beta \right)}e^{jkrsin\theta cos\left(\emptyset -\emptyset_{n}\right)}\right]$$ (3)

$${AF}_{cyl}=\sum_{n=1}^{N}I_{n}e^{\alpha_{n}}[b_{1}+b_{2}e^{j(kdcos\theta +\beta )}]e^{jkrsin\theta cos(\emptyset -\emptyset_{n})}$$ (4)

Equation 4 gives us the array factor for a two ring cylindrical array having ring radius *r* and the vertical separation *d*. In the proposed design, we have *M* number of such cylindrical arrays and a circular ring, therefore, by combining the array factors of circular and cylindrical array; we get the array factor of the proposed design as:

$$AF=AF_{0}+\sum_{m=1}^{M}\sum_{n=1}^{N}I_{mn^{e^{\alpha_{mn}}}}\left[b_{m1}+b_{m2e^{j\left(kh_{m}cos\theta +\beta_{m}\right)}}\right]e^{jkr_{msin\theta \text{cos}\left(\emptyset -\emptyset_{mn}\right)}}$$ (5)

Where *AF*_0_ is the array factor of the outer most circular ring placed at the x-y plane given as:

$$AF_{0}=\overset{N}{\underset{n=1}{\sum }}I_{n}e^{j\alpha_{n}}.e^{jkr_{0sin\theta \text{cos}(\left(\emptyset -\emptyset_{n}\right)}}$$ (6)

*N* is the total number of elements in a ring *M* is the total number of cylindrical arrays *h*_*m*_ is the vertical height between two rings of the *m* ^*th*^ cylindrical array *r*_*m*_ is radius of the rings of *mth* cylindrical array *I*_*mn*_,*α*_*mn*_ are the magnitude and phase excitations, respectively, of the *nth* element of the rings of the *m* ^*th*^ cylinder *β*_*m*_ is the linear phase excitation of the *m* ^*th*^ cylindrical array *b*_*m*1_ and *b*_*m*2_ are the magnitude excitation of the *nth* elements of the two rings of the *m* ^*th*^ cylindrical array along vertical direction. In the proposed array, these are taken as 1 for uniform magnitude excitation.

## Results and Discussions

In this section, simulation results are presented and compared with the existing cylindrical and coaxial cylindrical arrays. The important parameters such as directivity, HPBW and SLL of the proposed geometry are simulated. By varying the inter-element spacing, radii and number of elements on the circular rings, different results are plotted and compared.

### Beam Steering

The array factor expression for the proposed geometry as given in Equation (5) can be rewritten in its expanded form as:

$$AF=\overset{N}{\underset{n=1}{\sum }}I_{n}e^{jkr_{0}sin\theta cos\left(\emptyset -\emptyset_{n}\right)+\propto_{n}}+\overset{M}{\underset{m=1}{\sum }}\overset{2}{\underset{p=1}{\sum }}\overset{N}{\underset{n=1}{\sum }}I_{mn}e^{j\left(p-1\right)\left(kd_{m}cos\theta +\beta_{m}\right)}e^{jkr_{m}sin\theta cos\left(\emptyset -\emptyset_{mn}\right)}$$ (7)

**Fig 2:**
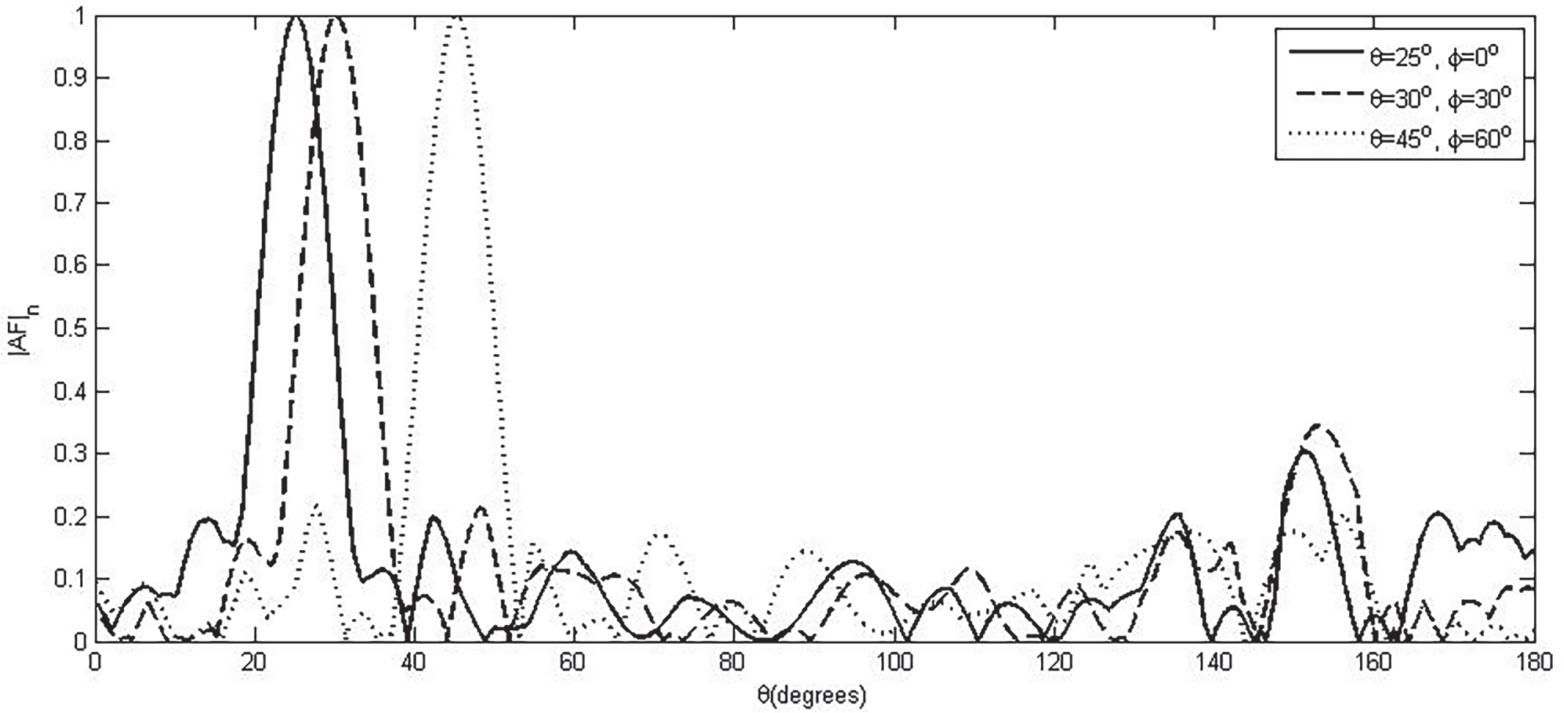
Beam Steering of Proposed Array.

By substituting the values of (*θ*_0_,*ϕ*_0_) in the above expressions, we can steer our beam in our desired direction. Table 1 shows an example of steering of main beam towards (*θ*_0_,*ϕ*_0_) with vertical spacing, *d*_*v*_ = 0.5λ, horizontal spacing, *d*_*r*_ = 0.5λ, number of elements in each 2-ring cylinder, *N* = 30 and number of cylinders, *M* = 3. The steering of the beams at three different steering angles is shown in Fig. 2. It can be noted that the radiation pattern of the array and hence the shape of the main beam remains almost unchanged with steering in the 3-D space. This is near to the ideal case of spherical array with much reduced overhead in the number of elements, size, and computation involved. In planar arrays and in cylindrical or coaxial cylindrical arrays, the main beam characteristics change significantly with steer angle in 3-D.

**Table 1:** Beam Steering.

Steer angle	Directivity (dB)	HPBW (degrees)	SLL (dB)
(*θ*_0_ = 25°,*ϕ*_0_ = 0°)	19.7335	7.4345	-6.906
(*θ*_0_ = 30°,*ϕ*_0_ = 30°)	19.6618	6.5800	-6.71
(*θ*_0_ = 45°,*ϕ*_0_ = 60°)	19.6930	6.9530	-6.623

Another advantage of the proposed design is that we do not have any second maximum at *π* – *θ* as it was in the case of other 1-D and 2-D geometries (i.e linear, rectangular and circular etc). The spurious radiation may occur while steering the main beam which can be removed by the use of circular to linear array transformation as described in [11]. The level of these spurious radiations is quite less than the other 3-D geometries like cylindrical and coaxial cylindrical. It is clear from Fig. 2 that there is only one maximum that occurs at (*θ*_0_,*ϕ*_0_) while steering the main beam and does not have any grating lobe at *π* – *θ*.

### Antenna Array Parameters

**Fig 3:**
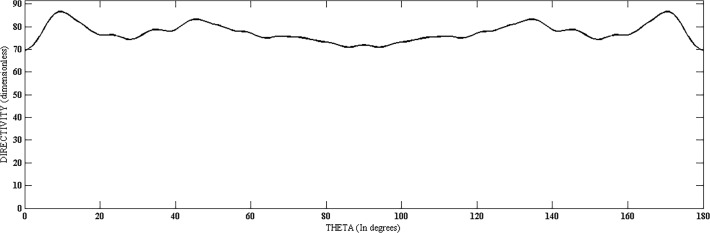
Directivity *vs*. Theta (at *ϕ* = 45°).

**Fig 4:**
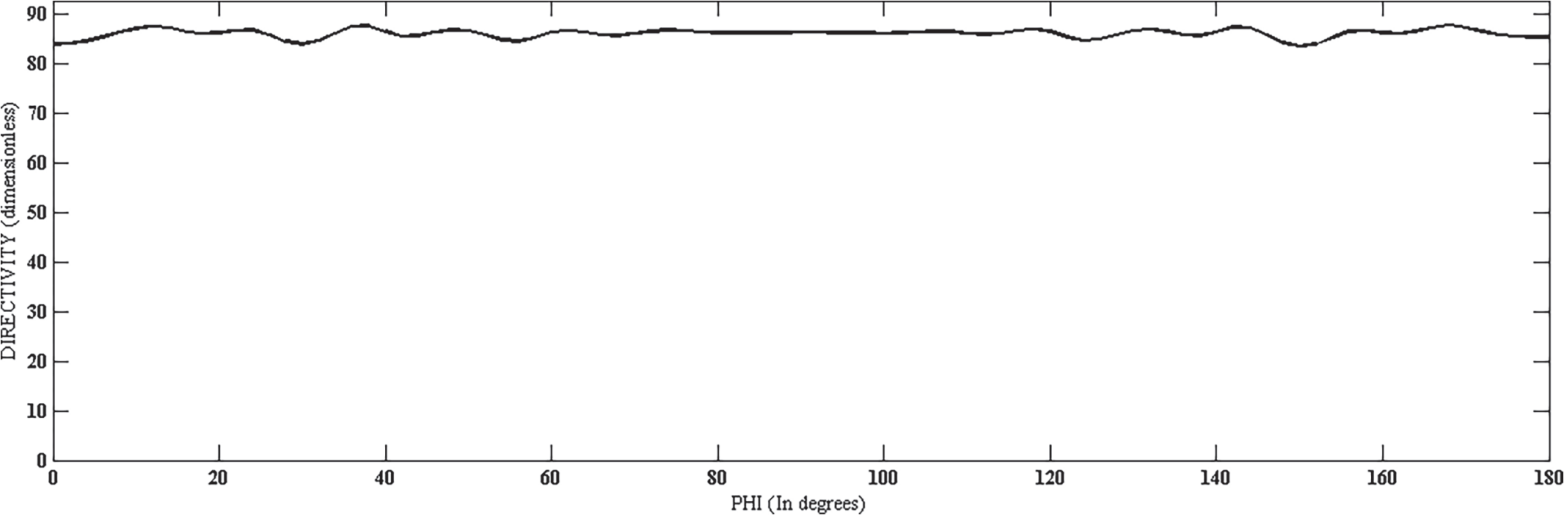
Directivity *vs*. Phi (at *θ* = 45°).

Directivity, HPBW and SLL of the proposed structure were evaluated at various steer angles. It was noted that the values of these parameters remain almost constant with slight variation, as shown in Fig. 2, 3, and 4 and Table 1 for few cases. Other parameters like N, N_cyl_, M and P are considered the same for different values of theta and phi. Fig. 3 and 4 are suggesting a uniform scanning along the azimuth and elevation, as the directivity remains almost constant with these angles.

**Fig 5:**
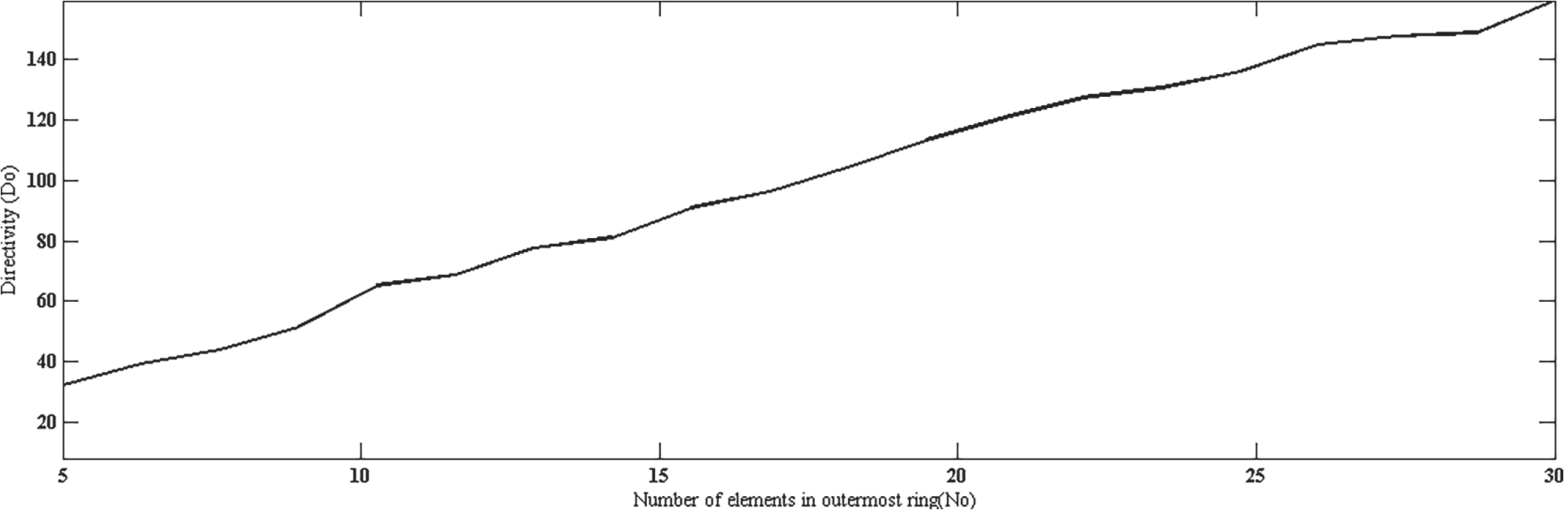
Directivity *vs*. Number of Elements in Outermost Ring.

**Fig 6:**
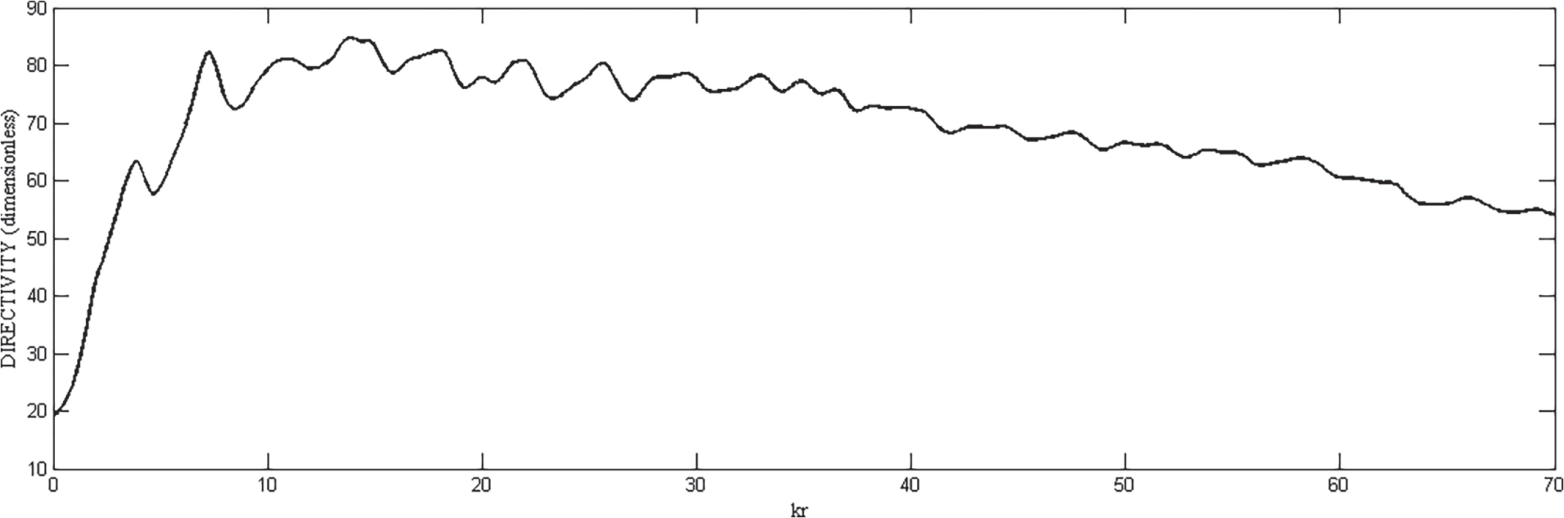
Directivity *vs*. *kr*.

**Fig 7:**
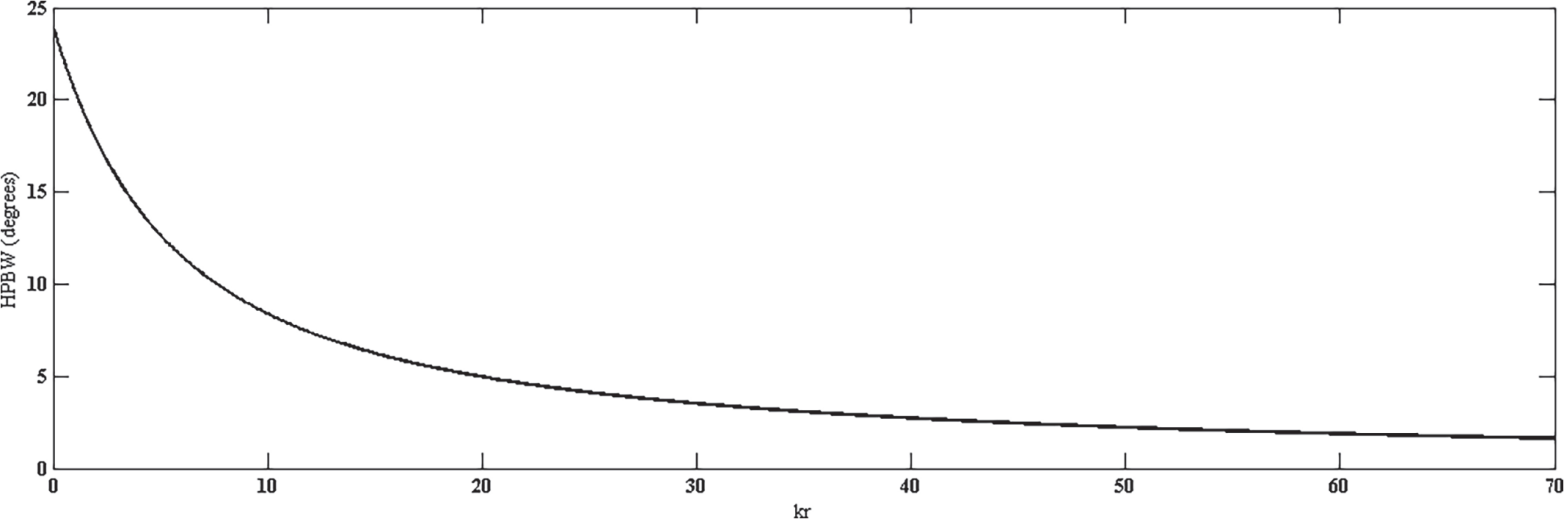
HPBW *vs*. *kr*.

**Fig 8:**
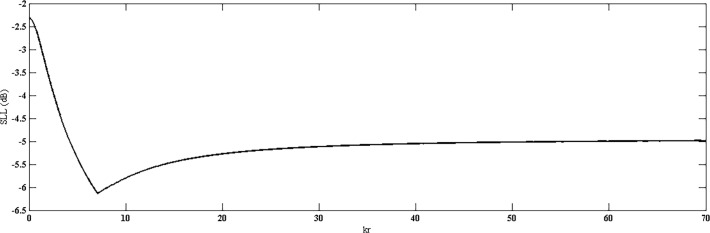
SLL *vs*. *kr*.

Directivity of the proposed geometry is plotted against the number of elements on the outermost ring, as shown in Fig. 5. Here, all the rings consist of same number of elements. The result shows that by increasing number of elements, higher values of directivity can be achieved. In Fig. 6, directivity is plotted against the radius *kr*. It can be seen clearly that with a fixed number of rings and elements, the directivity is poor with smaller radii in which the elements are placed more closely and hence mutual coupling can significantly affect the pattern. After a certain optimum spacing (which is half wavelength in most cases), the directivity becomes almost insensitive to the element spacing in the ring or the radius of the ring. Similar trends can be observed in HPBW and SLL. Both are high at closer element spacing due to mutual coupling and then reduce and remain almost constant with increase in radius. Fig. 7 and 8 suggest these results.

### Comparison with Other Geometries

**Fig 9:**
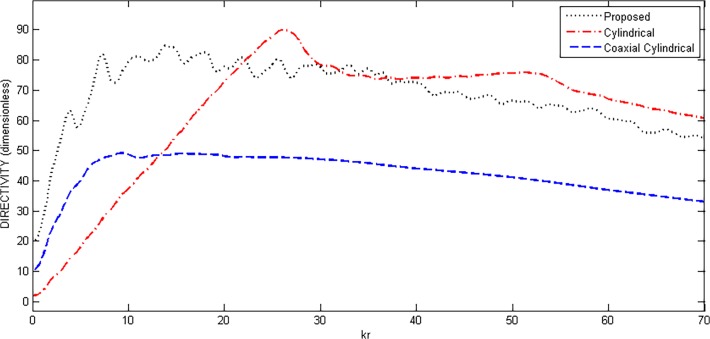
Directivity Comparison of Proposed, Cylindrical and Coaxial-Cylindrical Arrays Against Radius *kr* for *N* = 15, *N*_*cyl*_ = 45, M = 3, P = 2 at *θ*_0_ = 45°, *ϕ*_0_ = 45°.

**Fig 10:**
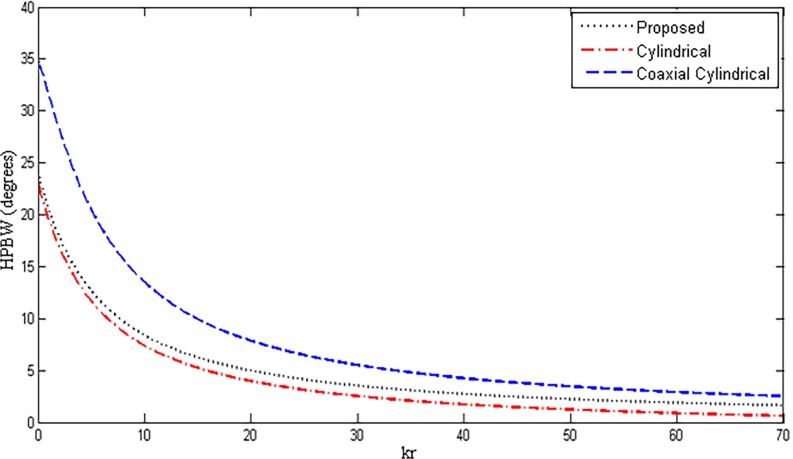
HPBW Comparison of Proposed, Cylindrical and Coaxial-Cylindrical Arrays Against Radius *kr* for *N* = 15, *N*_*cyl*_ = 45, M = 3, P = 2 at *θ*_0_ = 45°, *ϕ*_0_ = 45°.

**Fig 11:**
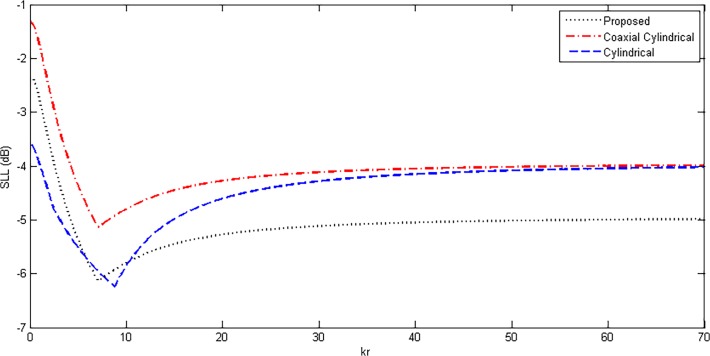
SLL Comparison of Proposed, Cylindrical and Coaxial-Cylindrical Arrays Against Radius *kr* for *N* = 15, *N*_*cyl*_ = 45, M = 3, P = 2 at *θ*_0_ = 45°, *ϕ*_0_ = 45°.

In this section, the values of directivity, HPBW and SLL are compared with the standard cylindrical and coaxial cylindrical array geometries. Regarding the 3-D case, double uniform linear array (DULA) and double uniform circular array (DUCA) possess good properties [15]. The disadvantage of these geometries is their size. For better values of directivities and other parameters, if the number of elements is increased, the size of overall array will also be increased accordingly, which is undesirable in most applications. The proposed array gives the same results with the reduced size. Also, the geometries of DUCA and DULA are already present in the proposed array due to its hybrid nature. These arrays can be formed by only changing the values of M and P in Equation (5). Cylindrical and coaxial cylindrical geometries have good properties as compared to previous 2-D and 3-D geometries [11–12], therefore, these geometries are chosen for comparison with the proposed array design. For fair comparison, some of the parameters of the three structures were fixed, shown in Table 2, while the radius *r* of the inner most ring of the array in each case was varied. The other parameters are varied accordingly with the inner ring, making it sure that the number of elements and the corresponding geometries of the three structures remain the same. Variations in directivity, HPBW and SLL were observed and plotted. Figs. 9–11 show the results of directivity, HPBW and SLL, respectively, against the radius *kr*.

**Table 2:** Parameters Of The Array Structures.

Parameter	Definition	Value
*N*	Number of elements in a circular ring of proposed array	*N* = 15
*N*_*cyl*_	Number of elements in a circular ring of cylindrical array	*N*_*cyl*_ = 45
*M*	Total number of cylinders in the proposed array	*M* = 3
*P*	Number of circular rings in a cylinder	*P* = 2
*d*_*v*_	Vertical spacing between two consecutive rings	*d*_*v*_ = 0.5λ
*d*_*r*_	Horizontal spacing between two consecutive rings	*d*_*r*_ = 0.5λ
(*θ*_0_,*ϕ*_0_)	Maximum scanning angles	(*θ*_0_ = 45°,*ϕ*_0_ = 45°)

It can be seen from Fig. 9 that the proposed geometry has shown better performance in directivity compared to the cylindrical and coaxial cylindrical array geometries. However, for cylindrical array, directivity has slightly higher values but for larger size with an extra grating lobe. In Fig. 10, HPBW is compared and plotted against radius *kr*. The results show that the proposed array has lower values of HPBW than the coaxial cylindrical geometry but very close to that of cylindrical array. The proposed array gives the best side lobe reduction compared to the other two comparable structures.

**Fig 12:**
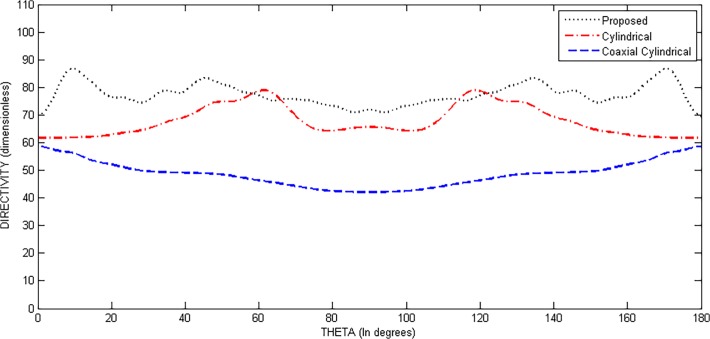
Directivity Comparison of Proposed, Cylindrical and Coaxial-Cylindrical Arrays Against *θ* for N = 15, *N*_*cyl*_ = 45, M = 3, P = 2 at *θ*_0_ = 45°, *ϕ*_0_ = 45°.

**Fig 13:**
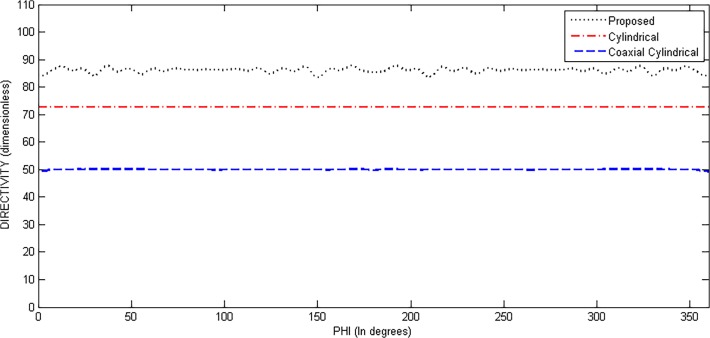
Directivity Comparison of Proposed, Cylindrical and Coaxial-Cylindrical Arrays Against *ϕ* for N = 15, *N*_*cyl*_ = 45, M = 3, P = 2 at *θ*_0_ = 45°, *ϕ*_0_ = 45°.

Fig. 12 and Fig. 13 show the values of directivity against *θ* and *ϕ*, respectively, for the three structures. In Fig. 12, we can see that the directivity has its maximum value for proposed geometry throughout the scanning process over *θ* while keeping *ϕ* fixed at 45°. Fig. 13 shows plot of directivity against *ϕ* while keeping *θ* fixed at 45°. The values of directivity are greater for proposed design as compared to the cylindrical and coaxial cylindrical arrays. Both these figures suggest a much uniform scanning in 3-D sphere by the proposed array.

## Conclusion

A 3-D antenna array structure is proposed which provides higher directivity and low SLL. The proposed structure gives uniform beam scanning in 3-D sphere with very less variation in its radiation pattern and main lobe characteristics with the scanning angle. The uniform scanning capability is provided by spherical arrays but with much more computational overhead due to its complex structure and large number of elements. The proposed structure achieves 3-D scanning close to the spherical array scanning with much reduced size and simple structure. The variation of directivity against *theta* and *phi* is almost constant in the proposed case as compared to the other structures. Moreover, the proposed structure provides a reduced level of side lobes compared to other comparable 2-D and 3-D structures and has no grating lobe, thus avoiding any false detection. The reduction in sidelobe level is up to 2 dB for different values of *kr*. Variations in directivities, HPBW and SLL have been calculated by changing different parameters of the array. The hybrid characteristics of the proposed geometry have been introduced in order to make a simple expression for the array factor.
